# Large Scale Association Analysis Identifies Three Susceptibility Loci for Coronary Artery Disease

**DOI:** 10.1371/journal.pone.0029427

**Published:** 2011-12-27

**Authors:** Stephanie Saade, Jean-Baptiste Cazier, Michella Ghassibe-Sabbagh, Sonia Youhanna, Danielle A. Badro, Yoichiro Kamatani, Jörg Hager, Joumana S. Yeretzian, Georges El-Khazen, Marc Haber, Angelique K. Salloum, Bouchra Douaihy, Raed Othman, Nabil Shasha, Samer Kabbani, Hamid El Bayeh, Elie Chammas, Martin Farrall, Dominique Gauguier, Daniel E. Platt, Pierre A. Zalloua, the FGENTCARD consortium

**Affiliations:** 1 School of Medicine, Lebanese American University, Beirut, Lebanon; 2 The Wellcome Trust Centre for Human Genetics, University of Oxford, Headington, Oxford, United Kingdom; 3 Centre d'Etude du Polymorphisme Humain (CEPH), Paris, France; 4 CEA-Genomics Institute, Centre National de Génotypage, Bât G2, Evry, France; 5 Brain Mind Institute, Ecole Polytechnique Fédérale de Lausanne, Lausanne, Switzerland; 6 Division of Cardiology, Rafic Hariri University Hospital, Beirut, Lebanon; 7 INSERM UMRS872, Centre de Recherche des Cordeliers, Paris, France; 8 IBM T. J. Watson Research Center, Yorktown Heights, New York, United States of America; 9 Harvard School of Public Health, Boston, Massachusetts, United States of America; University of Texas MD Anderson Cancer Center, United States of America

## Abstract

Genome wide association studies (GWAS) and their replications that have associated DNA variants with myocardial infarction (MI) and/or coronary artery disease (CAD) are predominantly based on populations of European or Eastern Asian descent. Replication of the most significantly associated polymorphisms in multiple populations with distinctive genetic backgrounds and lifestyles is crucial to the understanding of the pathophysiology of a multifactorial disease like CAD. We have used our Lebanese cohort to perform a replication study of nine previously identified CAD/MI susceptibility loci (*LTA*, *CDKN2A*-*CDKN2B*, *CELSR2*-*PSRC1*-*SORT1*, *CXCL12*, *MTHFD1L*, *WDR12*, *PCSK9*, *SH2B3*, and *SLC22A3*), and 88 genes in related phenotypes. The study was conducted on 2,002 patients with detailed demographic, clinical characteristics, and cardiac catheterization results. One marker, *rs6922269*, in *MTHFD1L* was significantly protective against MI (OR = 0.68, p = 0.0035), while the variant *rs4977574* in *CDKN2A*-*CDKN2B* was significantly associated with MI (OR = 1.33, p = 0.0086). Associations were detected after adjustment for family history of CAD, gender, hypertension, hyperlipidemia, diabetes, and smoking. The parallel study of 88 previously published genes in related phenotypes encompassed 20,225 markers, three quarters of which with imputed genotypes The study was based on our genome-wide genotype data set, with imputation across the whole genome to HapMap II release 22 using HapMap CEU population as a reference. Analysis was conducted on both the genotyped and imputed variants in the 88 regions covering selected genes. This approach replicated *HNRNPA3P1*-*CXCL12* association with CAD and identified new significant associations of *CDKAL1, ST6GAL1,* and *PTPRD* with CAD. Our study provides evidence for the importance of the multifactorial aspect of CAD/MI and describes genes predisposing to their etiology.

## Introduction

Given increased life expectancy, prevention of diseases with severe manifestations and later life complications has become of tremendous importance. Researchers' interest in identifying coronary artery disease (CAD) susceptibility genes reflects the very high mortality associated with both the disease [1] and one of its most serious complications: myocardial infarction (MI). While identifying the primary causes of CAD has been complicated by the multifactorial nature of the disease, genome-wide association studies (GWAS) have been moderately successful in revealing some of the polygenic structure of CAD. However, they only explain a modest fraction of the total heritability of CAD [2], [3].

Linkage disequilibrium is the primary mechanism for generating direct (causal) and indirect (non-causal) genetic associations. Population stratification following population mixing [4], [5] can lead to large numbers of correlated mutations [5], [6], [7], almost all of which are expected to be non-causal [8]. Population effects may also produce strong associations in some populations but not others by means of specific enrichment due to population genetic effects (e.g. drift and founder effect) [4], [5], [9], [10]. Furthermore, in view of the multifactorial nature of CAD, the effects of genes acting as component causes may be discernible in some populations but not in others depending on diet, lifestyle, and genetic background. Recent GWAS and their replications that have established highly significant associations of variants with MI and/or CAD are predominantly based on populations of European [11], [12], [13], Eastern Asian [11], [14], [15], and more recently, South Asian descent [16]. Replication of these results for the most significantly associated polymorphisms in multiple populations with distinguishing genetic and lifestyle backgrounds is crucial in isolating the pathophysiology of a multifactorial disease like CAD.

Here, in complement to a GWAS performed for MI and CAD on the same subjects, we have sought to perform a replication study in a Middle-Eastern population of SNPs identified in GWAS of other populations. We have selected susceptibility loci based on prior studies to confirm the association of 10 recently identified SNPs at nine independent loci (Table 1) associated with increased risk of CAD and/or MI in 4 major GWAS conducted in populations of European [11], [12], [13] and East Asian ancestry [11], [14]. In parallel, we have assessed association with CAD phenotype at 88 genes associated with related phenotype using patterns of correlation among SNPs from the International HapMap project around 4,784 genotyped SNPs from these candidate regions to impute genotype at 15,441 collocated, but unavailable marker.

**Table 1:** Description of the 10 SNPs used in the study.

Chr / rs	Gene	Association with	Risk allele	Population site	Reference
6p21 / *rs1041981*	*LTA*	MI	*A*	Japan	Ozaki et al., 2002
9p21 / *rs4977574*	*CDKN2A-CDKN2B*	MI	*G*	Italy	Kathiresan et al., 2009
1p13 / *rs646776*	*CELSR2*-*PSRC1*-*SORT1*	MI	*T*	US	Kathiresan et al., 2009
1q41 / *rs1746048*	*CXCL12*	MI	*C*	Spain	Kathiresan et al., 2009
6q25 / *rs6922269*	*MTHFD1L*	MI	*A*	Finland	Kathiresan et al., 2009
2q33 / *rs6725887*	*WDR12*	MI	*C*	Sweden	Kathiresan et al., 2009
1p32 / *rs11206510*	*PCSK9*	MI	*T*		Kathiresan et al., 2009
12q24 / *rs3184504*	*SH2B3*	MI	*T*	Iceland Europe	Gudbjartsson et al., 2009
12q24 / *rs653178*	*SH2B3*	MI	*C*	Asia	Gudbjartsson et al., 2009
6q26-27 / *rs2048327*	*SLC22A3*	CAD	*C*	Britain Germany	Trégouët et al., 2009

For each SNP, genomic location, gene site, association with CAD and/or MI, risk allele, and populations in which association was found are displayed in left to right columns. For each association study, reference is indicated.

Abbreviations: Chr, chromosome; SNP, single nucleotide polymorphism; MI, myocardial infarction; CAD, coronary artery disease.

## Materials and Methods

### Study Subjects

A total of 2,002 subjects recruited between 2006 and 2009 for inclusion in the FGENTCARD database (www.well.ox.ac.uk/fgentcard) were selected. These subjects were referred to a catheterization care unit for clinical evaluation. The 4 main coronary arteries: the left main artery (LMCA), the left anterior descending artery (LAD), the left circumflex artery (LCx), and the right coronary artery (RCA) were visualized from different angles by angiography. The extent of stenosis in these vessels was assessed and recorded by percentage. Some of these patients were admitted to the hospital for having an MI as diagnosed by electrocardiogram and high troponin levels. Cardiologists performing the coronary angiography collected a 20 mL blood sample from the peripheral femoral artery of patients who provided a written consent for the whole study including the genetic analysis. Blood chemistry metrics were obtained. Trained healthcare professionals collected further data on the socio-demographic background of the patients. Annotations were coded from medical charts according to our study protocol, which included results of any additional data such as laboratory tests, prescribed medications, and presence of other diseases and conditions. Physician classified a patient as dyslipidemic when LDL>130 mg/dl and HDL<40 mg/dl, diabetic (type 2) if fasting glucose level>125 mg/dl, hypertensive when blood pressure was >10/14 mm Hg. A positive family history was defined as a sibling, parent, or second-degree relative with a coronary event.

Genomic DNA was extracted using a standard phenol extraction protocol. Genotyping was performed on Illumina Human610-Quad and Illumina Human660W-Quad BeadChips, at the Centre National de Génotypage, France. Out of the 2,002 genomic DNA samples analyzed, 1,949 non-duplicated samples with quality filtered genotyping data were retained. After the removal of three individuals with genotyping success rate less than 95% and 19 pairs of individuals which were suspected to have been duplicated, 1,949 individuals with quality filtered genotyping data were retained. Genotyping success rate was more than 98%, minor allele frequency more than 1%, and the *p* value of Hardy-Weinberg equilibrium test was less than 1×10^−7^. The Institutional Review Board at the Lebanese American University approved the study protocol.

### Statistical analyses of the nine candidate loci

Our study is a retrospective cross-sectional design with subjects drawn from catheterized patients. Two clinical outcomes were considered in this phase of the study: coronary artery stenosis and MI. In the coronary artery stenosis outcome, subjects were categorized as those with no sign of stenosis (“super-controls”, CAD category 1, n = 425), whereas cases were patients categorized into two classes [17] CAD category 2 for patients with stenosis of less than 50% in at least one vessel (n = 214), and CAD category 3 for patients with stenosis greater than 50% in at least one vessel diagnosed by catheterization (n = 1,310). MI diagnosis was established based on medical examination upon admission to the hospital. In the MI outcome, cases were patients admitted to the hospital with MI positive (n = 222) whereas controls (n = 1,727) were patients admitted for reasons other than MI, such as unstable angina or cardiac diagnostic workup.

We addressed the effects of conventional risk factors for coronary artery. We determined the Chi-squared significance for categorical variables: gender, presence of diabetes mellitus, hypertension, smoking, and family history of CAD, and Welch *t*-test for continuous variables: FBG, total cholesterol, HDL, LDL, triglycerides, age, and body mass index (BMI). Categorical variables were summarized as percentages while continuous variables with parametric distributions were summarized as means and standard deviations. Age of onset was expressed as median and interquartile range (IQR) and triglyceride levels were logarithmically scaled before analysis (Table 2). Statistical differences between controls and cases for these risk factors were assessed using the unpaired two-sided *t*-test for the continuous variables and the chi-square test for categorical variables (Table 2).

**Table 2:** Distribution of biological and biochemical characteristics of patients by stenosis category (n = 1,949).

	Total (n = 1,949)	No stenosis (n = 425)	Stenosis (n = 1,524)	p-value
**Males (%)**	72.7	57	77.1	2.4×10^−16^
**Diabetes (%)**	29.7	16.2	33.4	9.5×10^−13^
**Hypertension (%)**	54.6	43	58	4.6×10^−8^
**Hyperlipidemia (%)**	47.9	36.2	51.2	3.8×10^−8^
**Smoking (%)**	64.4	55.6	66.9	2.8×10^−5^
**Family History of CAD (%)**	61.8	65.4	56.1	0.0059
**Age of onset (Median ±IQR)**	--	--	62 ±16	--
**Glucose (mg/dl)**	118.8 ±42.5	106.9 ±33.7	122.0 ±44.1	2.6×10^−11^
**Total Cholesterol (mg/dl)**	187.1 ±48.4	187.7 ±42.2	188.0 ±49.9	0.925
**HDL (mg/dl)**	41.3 ±12.0	42.6 ±13.0	41.0 ±11.7	0.029
**LDL (mg/dl)**	114.2 ±41.1	114.1 ±34.2	114.2 ±42.7	0.95
**Log (triglycerides)**	5.1 ±0.5	5.0 ±0.5	5.1 ±0.5	0.15
**BMI**	29.0 ±4.7	29.4 ±5.2	28.9 ±4.6	0.11

Left column lists the risk factors investigated upon recruitment of the study population. Averages values of biochemical characteristics are indicated for each of the total population, population without stenosis, and population with stenosis. The P-value is the chi-square.

The 10 SNPs analyzed (Table 1) were selected based on their association with CAD/MI and were searched for using the NCBI PubMed Entrez database (http://www.ncbi.nlm.nih.gov/sites/gquery). The association of each SNP with CAD and/or MI was assessed in genotypic and allelic association models using logistic regression.

Proportional odds logistic regression was performed across CAD categories, and standard logistic regression was performed on MI cases vs. controls. The candidate risk alleles were categorized as absent (allele score = 0), heterozygous (allele score = 1), and homozygous (allele score = 2). Logistic regression analyses treated the allele score linearly, corresponding to treating the genotype heterozygous vs. homozygous states as additive to risk, and in separate analyses, treating each allele score as distinct risk categories. Regressions were performed for unadjusted risk odds ratio estimates, as well as adjusted analyses for family history of CAD, gender, hypertension, hyperlipidemia, diabetes, and smoking.

### Imputation-based analysis of candidate genes

Coronary artery stenosis was the only clinical outcome considered in this phase of the study. A total of 20,225 variants located in 88 different genes were tested for association in our Lebanese CAD cohort using imputation-based analysis. SNPs were selected on the basis of MI/CAD phenotype in NCBI Phenotype-Genotype Integrator (PheGenI) (http://www.ncbi.nlm.nih.gov/gap/PheGenI#pgGAP) and on literature review [16], [18], [19], [20]. Imputation utilizes the Lebanese population genotype data from 4,784 SNPs, in combination with haplotype information from the CEU population of the release 22 of HapMap to predict genotypes of 15,441 nearby SNPs not genotyped in our subjects. Although the Principal Component Analysis identified our cohort to be closest to the HapMap CEU (data not shown), some underlying different genetic background with the discovery cohorts may exist. We therefore tested for association across the corresponding 88 genes rather than individual variants (Table S1). After imputing across the whole genome to HapMap CEU reference population using Impute v2 [21], we selected both the genotyped and imputed variants in the 88 regions covering the selected genes, allowing for an extra 50 Kb on either side. Because of the probabilistic nature of the genotypes inferred by imputation, we applied SNPTest2 to perform an additive association test using a missing data likelihood score ("-frequentist 1 -method score"), including age and sex as covariates [22]. Effect and significance were reported for association with p-value<0.01 and marker with info>0.4 (Table S1).

## Results

Following QC filtering, the sample consisted of 1,949 subjects, 72.7% of whom were males, with a median ±IQR age of 62±16 years, and a mean BMI of 29.0±4.7 kg/m^2^. Among these subjects, 54.6% were hypertensive, 48.0% were hyperlipidemic, 61.8% had a family history of CAD (parents or siblings), and 29.7% were diabetic. Moreover, higher prevalence among cases compared to controls of diabetes, hypertension, hyperlipidemia, family history of CAD, raised glucose levels, and reduced HDL levels, were observed (Table 2).

The probability of 2 or fewer successes out of 10 Bernoulli trials at the 0.05 significance threshold is 0.0861. However, minor allele frequencies were sufficiently low among a number of SNPs for cases or controls that information about those specific counts was compromised. Only the MI test for *rs4977574* was significant after over conservative Bonferroni correction for a one-tail test, while only the *rs6922269* was found significantly protective after Bonferroni correction for CAD.

Logistic regressions testing 600K SNP PCA k-means clusters (k = 4), capturing extremes of the leading Lebanese-specific principal components, did not predict MI or CAD, nor interact with risk factors or identified risk SNPs in the prediction of CAD or MI (data not shown). Population stratification identified in the cohort was shown not to have a significant effect in the association with a genomic control of λ =  1.033 (data not shown).

Risks associated with each allele expressed as odds ratios, odds ratios adjusted for gender, hypertension, hyperlipidemia, smoking, and diabetes, are displayed in Table S2 for CAD, and Table S3 for MI. Eight of the ten SNPs, *rs1041981*, *rs11206510*, *rs1746048*, *rs3184504*, *rs646776*, *rs653178*, *rs6725887*, and *rs2048327*, showed no two-tailed significant associations with CAD or MI (Tables S2 and S3).

SNP *rs4977574* was not significantly associated with CAD (Table S2), but was significantly associated with MI in a proportional odds logistic regression (OR = 1.86, 95%CI = 1.17−3.07, p = 0.012) for the *GG* genotype compared to the *AA* genotype (Table S3). In an adjusted analysis, this association with MI was unaltered (OR = 1.84, 95%CI = 1.14−3.09, p = 0.011) (Table S3). The heterozygous *GA* genotype vs. *AA* showed odds ratios of 1.39 (unadjusted) and 1.42 (adjusted), but were insignificant (Table S3). Allele frequency results were also insignificant contrasting *G* vs. *A* alleles (OR = 1.38, 95%CI = 1.01−1.90, p = 0.039). All adjustment risk factors made highly significant contributions to the proportional odds logistic regression (Table 3).

**Table 3:** Association of CAD with *rs497754* adjusting for each of gender, hypertension, hyperlipidemia, smoking, and diabetes.

Interactive Risk	Adj. OR 95% CI	p-value *power*
**Gender** (Female as reference)	2.511 (2.00, 3.15)	<0.0001 *1.0000*
*GG*	1.403	0.0205
	(1.01, 1.94)	*0.533*
*AG*	1.269	0.0692
	(0.93, 1.74)	*0.316*
*AA*		
**DxHypertension**	1.834	<0.0001
	(1.48,2.28)	*0.9998*
*GG*	1.423	0.0161
	(1.03, 1.96)	*0.573*
*AG*	1.252	0.0805
	(0.91, 1.71)	*0.288*
*AA*		
**DxHyperlipidemia**	1.845	<0.0001
	(1.48, 2.30)	*0.9997*
*GG*	1.359	0.0304
	(0.98, 1.87)	*0.466*
*AG*	1.24	0.0887
	(0.91, 1.69)	*0.271*
*AA*		
**Smoking**	1.636	<0.0001
	(1.31,2.04)	*0.993*
*GG*	1.337	0.0377
	(0.97, 1.84)	*0.428*
*AG*	1.202	0.124
	(0.88, 1.64)	*0.21*
*AA*		
**DxDiabetes**	2.466	<0.0001
	(1.87, 3.25)	*1.0000*
*GG*	1.402	0.0198
	(1.02, 1.93)	*0.539*
*AG*	1.228	0.0991
	(1.86 3.25)	*0.025*
*AA*		

Abbreviations: Adj., adjusted; OR, odds ratio; CI, confidence interval.

The variant *rs6922269*, was seen to be robustly significant as protective against MI. The heterozygous odds ratio was highly significant (OR = 0.66, 95%CI = 0.48−0.9, p = 0.0087), and remained significant after adjustment (OR = 0.63, 95%CI = 0.45−0.85, p = 0.0037) (Table S3). The additive model risk showed an OR = 0.72, 95%CI = 0.56−0.91, p = 0.0083, and adjusted OR = 0.69, 95%CI = 0.53−0.88, p = 0.0035. It is interesting to note that the measured OR for the homozygous allele was 0.63, 95%CI = 0.31−1.15, p = 0.163 before adjustment, and OR = 0.60, 95%CI = 0.30−1.10, p = 0.121 after adjustment, though while as strong as the heterozygous risk, the error bars were much wider suggesting the population size of n = 11 for MI subjects carrying the homozygous allele was too small to resolve this risk, also suggestive of a protective power for the allele.

Analysis of our Illumina data allowed us to query much of the known SNP variations in the genome. Using an imputation method as a thorough survey of the variations from 88 genes, a total of 15,441 SNPs were imputed genotypes in addition to the 4,784 genotyped SNPs. Table S1 presents an overview of the most nominally significant SNPs (*p*<0.01) in the regions that have been reported for CAD. SNPs located in the linked regions of *CDKAL12*, *ST6GAL1*, *CXCL12*, and *PTPRD* were the most highly associated with *p* values <0.0009. Rs9295489 in the *CDKAL1* locus presented the highest association with CAD (beta coefficient = 0.12, p = 0.0004) (Table S1). The locus increased the risk of CAD of about 1.12. Three imputed SNPs in the 50 Kb block of a second locus, *ST6GAL1*, had *p* values ranging from 0.0007 (rs16861460) to 0.0008 (rs16861471). The locus had a protective effect against CAD with an estimated beta coefficient of −0.17, corresponding to an odds ratio of 0.8 (Table S1). A third gene, *CXCL12*, was found to play a role in CAD etiology. The SNP rs7900896 (beta coefficient = 0.12, p = 0.0008) located in the *CXCL12* LD block gave high relative information about the additive model and showed significant association with CAD. A fourth LD region defining the *PTPRD* locus had one in the top six hits of our analysis (rs10115782; beta coefficient = 0.2, p = 0.0009). It was highly informative and presented significant association with CAD (Table S1).

## Discussion

Replication studies are subject to variations in design adding a significant level of complication to comparative analysis [23]. Variability in study enrollment, as well as the use of different inclusion and exclusion criteria such as age of onset, all have the potential to impact risk estimates [12], [24]. Most importantly, CAD outcomes are classified by varying standards. While some use coronary angiography to firmly verify the disease status and to determine whether a patient should belong to the cases or controls groups, others are considering apparently healthy individuals from the general population as controls. A major strength of our study relies on the unambiguous angiographic visualization of CAD phenotypic characterization. A longitudinal prospective study sampling the population at large would have been ideal to increase the frequency of some genotypes and exclude any risk of bias consequent to low frequency representation. However such invasive measures of disease would be ethically impossible.

We have found that the variant *rs4977574* located within the 9p21 locus confers an increased risk of developing MI in the *GG* state. Moreover, subjects homozygous for the *G* allele are at a much higher risk of MI. These results are in accordance with a study by Kathiresan where *rs4977574 G* allele was shown to be associated with MI [12]. This study argued that altered transcript levels of *CDKN2B* in visceral tissue may increase the risk of atherosclerosis emergence [12]. It is pertinent to note that since our study and the GWAS mentioned are all retrospective studies, the *rs4977574* risk was survived by patients in treatment of CAD and/or MI long enough to be enrolled in the study.

It is of importance to note that the 9p21 locus, in particular variant *rs4977574*, did not show genome wide significance for CAD (p = 0.0107) or MI (p = 0.00448) when a GWAS was conducted on this study population (data not shown). This SNP is within a range consistent with the logistic regression results, though not significant after over-conservative Bonferroni correction.

The *rs4977574* variant is located within the *ANRIL* gene that encodes a large non-coding RNA [25]. While it still needs to be elucidated, implication of *ANRIL* in the pathophysiology of coronary artery syndrome has been strongly supported by expression studies [26], [27], [28]. The *ANRIL* promoter region contains binding sites for zinc-finger proteins that are critical for the transcription of *CDKN2A* and *CDKN2B* [29]. In the mouse, deletion of the 9p21 orthologous non-coding region including the 3′ region of *ANRIL* affects proliferation of vascular cells while expression of *CDKN2A* and *CDKN2B* is severely reduced [30] thus indicating potential implications in the disease etiology in humans.

The variant *rs6922269* has shown a protective effect against MI in our population. Association of this variant was already characterized by others [31] through analysis of the Wellcome Trust Case Control Consortium (WTCCC) [32] population. However this association was not confirmed in the Tunisian population [33], and functional implications of the gene in CAD etiology have not been investigated. It can be hypothesized that the inverse relationship of *rs6922269* compared to other studies suggests significant interactions with other exposure, or an inverted linkage with the active alleles in the Lebanese population relative to other populations showing risk.

None of the remaining eight gene variants identified as significant CAD or MI risks in other populations showed a contribution to risk of CAD or MI in the Lebanese population, even though some of them are implicated as candidates through genetic association [11], [12] and molecular mechanisms [34], [35], [36], [37], [38], [39]. The negative results regarding these SNPs are important to consider since the differences in association across populations could indicate differences due to lifestyle or environment, or population-specific mutation interaction factors, or linkage variations mitigating disease development. For a number of these alleles, low power due to small numbers, especially in the reference category, may account for some of the losses of significance.

High-throughput genotyping chips such as the Illumina 660Hap used here provide a very good genome-wide coverage via tagSNPs which best represent the underlying haplotypes. This should allow for the identification of regions associated with given phenotypes. However, the tagSNPs provide statistical rather than functional association. It is therefore important to go beyond the original chip and look for the causative variant. Despite a potentially lower MAF, causative variants should have a stronger effect on the phenotype, and more biological relevance. Furthermore, in the case of replication of association originally observed in Caucasian, it is possible that the reported SNP is not associated in our Lebanese cohort. Since our population is sufficiently closely related to the CEU, we could use HapMap catalogue of haplotypes to impute the genotypes of all the markers in regions of interest to test optimally for association between the gene and the phenotype.

This approach unraveled positive association of CAD with *CXCL12*, a gene that maps to 10q11. A *CXCL12* intergenic SNP, rs1746048, had a C-risk allele associated with MI (OR = 1.17, p = 7×10^−9^) [12]. The same rs1746048-C allele yielded positive association results with CAD in a recent study (OR = 1.09, p = 3×10^−10^) [20]. Although rs1746048 was not associated with CAD in the Lebanese cohort, another SNP located in the same LD block, rs7900896, showed positive association results. The two SNPs are located 5′ of *CXCL12*, 467 Kb away. They are not in the same HapMap, CEU-based, LD block. Our results imply that this genomic region contains several causative variants. Whether the associated SNPs are predisposing by themselves, or whether they are in LD with the real variant needs further investigations.

Another SNP, rs9295489 in *CDKAL1*, showed positive association with CAD in our cohort. As a part of the Cardiovascular Health Study (CHS) GWAS that aims to identify genetic variants associated with cardiovascular risk factors (http://www.ncbi.nlm.nih.gov/projects/gap/cgi-bin/study.cgi?study_id=phs000226.v2.p1), rs2206734 in *CDKAL1* was associated with MI occurrence (p = 5.05×10^−4^). The two SNPs are intragenic, 345 Kb away, defining separate LD blocks. While our study confirms that variants near *CDKAL1* are associated with risk for cardiovascular anomalies, no published data has associated *CDKAL1* with CAD phenotype. Interestingly, since 2007, numerous studies have associated *CDKAL1* SNPs with a cardiovascular disease (CVD) risk factor: Type 2 Diabetes (T2D) [19], [40], [41], [42], [43]. Thus, rs9295489 in *CDKAL1* may influence T2D levels and consequently affect the risk for CVD.

Two genes, *ST6GAL1* and *PTPRD* (See Table S1 for positive SNPs), were shown to be associated with CAD for the first time in our study. Both genes are known to carry common variants predisposing to Diabetes Mellitus [18], [19], [44], [45]. Rs16861329 in *ST6GAL1* locus was associated with T2D and pancreatic beta-cell function [18]. The fact that in our study *ST6GAL1* has a protective effect on CAD occurrence suggests an inverted linkage between the active alleles or different alleles arising in various impacts on gene function, and thus, on health. *PTPRD*, previously shown to be a susceptibility locus for T2D [19], [44], [45], carries variants predisposing to CAD in the Lebanese cohort, showing again a genetic link between T2D and CAD.

A Bonferroni correction per interrogated marker (20,224) would be very over conservative since imputation identified many markers in perfect LD to be tested. A correction for the number of regions tested (88) would be more appropriate, and would retain the significance of these results even though these regions can encompass independent variants. We therefore consider these candidate genes to be of strong interest, but that they require further validation.

This association study investigates genetic factors underlying CAD/MI in individuals of Lebanese ancestry, a population with increased susceptibility to CAD. We identified common genetic variants at two loci previously identified as CAD/MI genetic risk factors (9p21 and *CXCL12*), and two loci newly associated with CAD (*CDKAL1* and *PTPRD*). We identified *MTHFD1L* and *ST6GAL1* as having a protective effect against CAD/MI. Cardiovascular diseases coexist with metabolic risk factors including central obesity and diabetes. Our study provides new insights into the genetic link between metabolic and cardiovascular traits. Some of the identified loci have implicated metabolic genes without a previously known connection with CAD. Future studies will build on these successes by identifying additional variants and by determining the functional impact of the underlying genes. Further, our findings show the potential for new discovery from genetic association studies in populations of non-European ancestry.

## Supporting Information

Table S1 **Variants' imputed genotype probabilities**. Imputed genotype probabilities for variants in 88 candidate genes with +/- 50Kb on each side. The test was considered significant when *P*<0.01, relative information >0.4 and minor allele frequency >5%.
(DOC)

Table S2 **Proportional Odds Logistic regression predicting CAD in graded categories from 10 SNPs.** Proportional Odds Logistic regression predicting CAD in graded categories from 10 SNPs, both with additive and independent homozygous/heterozygous odds, without and with adjustment by family history of CAD, history of smoking, diagnoses of diabetes, hyperlipidemia, hypertension, and gender are represented. Odds ratio tests of disease vs. haplotype frequency are also indicated.
(DOC)

Table S3 **Logistic regression predicting MI from 10 SNPs.** Analysis was performed both with additive and independent homozygous/heterozygous odds, without and with adjustment by family history of CAD, history of smoking, diagnoses of diabetes, hyperlipidemia, hypertension, and gender. Odds ratio tests of disease vs. haplotype frequency are also represented.
(DOC)
