# Supplementary material for: Large Scale Association Analysis Identifies Three Susceptibility Loci for Coronary Artery Disease
Source: PLoS One. 2011 Dec 27;6(12):e29427. doi: 10.1371/journal.pone.0029427 (PMC3246490; doi:10.1371/journal.pone.0029427)
Supplement: Table S1 — Variants' imputed genotype probabilities. Imputed genotype probabilities for variants in 88 candidate genes with +/- 50Kb on each side. The test was considered significant when P<0.01, relative information >0.4 and minor allele frequency >5%. (DOC) [file pone.0029427.s001.doc]

**Table S1. Variants’ imputed genotype probabilities. Imputed genotype probabilities for variants in 88 candidate genes with +/- 50Kb on each side. The test was considered significant when *P*<0.01, relative information >0.4 and minor allele frequency >5%.**

| **Gene** | **SNP id** | **Position** | ***P* value** | **Relative information about the model parameters** | **Estimates of the model parameters** | **Standard Error** |
| --- | --- | --- | --- | --- | --- | --- |
| CDKAL1 | rs9295489 | 21147930 | 0.0004612 | 0.9736 | 0.11137 | 0.031798 |
| ST6GAL1 | rs16861460 | 188206638 | 0.00073302 | 0.96687 | -0.17392 | 0.051501 |
| ST6GAL1 | rs16861456 | 188206305 | 0.00074211 | 0.95856 | -0.17485 | 0.051831 |
| ST6GAL1 | rs16861471 | 188207371 | 0.00080816 | 0.9874 | -0.17081 | 0.050988 |
| HNRNPA3P1 | rs7900896 | 43628006 | 0.00087179 | 0.90443 | 0.12472 | 0.037466 |
| PTPRD | rs10115782 | 8356629 | 0.0009996 | 0.80804 | 0.20852 | 0.063367 |
| CDKAL1 | rs9465941 | 21051211 | 0.0010795 | 1 | -0.1496 | 0.045765 |
| PTPRD | rs10976997 | 8356011 | 0.0011531 | 0.79521 | -0.20809 | 0.064023 |
| KCNQ1 | rs2079046 | 2455254 | 0.0012835 | 0.97783 | -0.2073 | 0.064387 |
| PTPRD | rs10815837 | 8358226 | 0.001339 | 0.86003 | -0.15538 | 0.048443 |
| PTPRD | rs10815836 | 8358178 | 0.0013417 | 0.86146 | 0.15541 | 0.048461 |
| PTPRD | rs10977004 | 8357920 | 0.0013647 | 0.87109 | 0.15532 | 0.048507 |
| PTPRD | rs10815835 | 8357899 | 0.001369 | 0.872 | -0.15528 | 0.048507 |
| PTPRD | rs10815838 | 8358465 | 0.0013771 | 0.85457 | -0.15455 | 0.048307 |
| PTPRD | rs10758962 | 8357697 | 0.0014111 | 0.88201 | 0.15477 | 0.048481 |
| HNRNPA3P1 | rs11238723 | 43635586 | 0.0014299 | 1 | 0.18284 | 0.057343 |
| ST6GAL1 | rs16861499 | 188215649 | 0.0014362 | 0.93879 | 0.16694 | 0.052377 |
| ADAMTS9 | rs4488822 | 64457749 | 0.0014739 | 1 | 0.11162 | 0.035102 |
| PTPRD | rs10977003 | 8356944 | 0.001712 | 0.93721 | -0.15047 | 0.047979 |
| CDKAL1 | rs9350305 | 21056472 | 0.0017167 | 0.98676 | -0.14086 | 0.044926 |
| CDKAL1 | rs7749498 | 21061898 | 0.0017362 | 0.98448 | -0.14059 | 0.04489 |
| HNRNPA3P1 | rs7920802 | 43627723 | 0.0017824 | 0.9693 | 0.15522 | 0.049681 |
| HNRNPA3P1 | rs7913725 | 43634437 | 0.0018058 | 1 | -0.18026 | 0.057767 |
| CDKAL1 | rs9295494 | 21239291 | 0.0019287 | 1 | 0.10135 | 0.032682 |
| ST6GAL1 | rs6791737 | 188207247 | 0.001963 | 1 | -0.13877 | 0.044827 |
| CDKAL1 | rs6456388 | 21068911 | 0.0019749 | 0.98382 | 0.1383 | 0.0447 |
| CDKAL1 | rs6915209 | 21071587 | 0.0020173 | 0.98394 | -0.13793 | 0.04467 |
| CDKAL1 | rs6941808 | 21072662 | 0.0020378 | 0.98408 | 0.13775 | 0.044656 |
| HNRNPA3P1 | rs2863234 | 43641162 | 0.0020437 | 0.93716 | -0.19973 | 0.064766 |
| DMRTA1 | rs538247 | 22440566 | 0.0021173 | 1 | 0.12186 | 0.039653 |
| CDKAL1 | rs7746754 | 21077716 | 0.0021183 | 0.98509 | -0.13709 | 0.044609 |
| PTPRD | rs10976999 | 8356231 | 0.0021593 | 0.94145 | 0.1477 | 0.048153 |
| PTPRD | rs10977013 | 8363105 | 0.0021595 | 1 | 0.12781 | 0.041666 |
| HNRNPA3P1 | rs7906426 | 43627613 | 0.0021705 | 1 | -0.14918 | 0.048659 |
| CDKAL1 | rs201358 | 21085653 | 0.0021912 | 0.98726 | 0.13653 | 0.044574 |
| CDKAL1 | rs201357 | 21087560 | 0.0022071 | 0.98786 | -0.13641 | 0.044566 |
| CDKAL1 | rs444005 | 21089616 | 0.0022203 | 0.98831 | 0.13632 | 0.044562 |
| CDKAL1 | rs201354 | 21093317 | 0.0022482 | 0.9875 | 0.13617 | 0.044568 |
| CDKAL1 | rs201351 | 21094109 | 0.002255 | 0.98737 | -0.13613 | 0.044569 |
| CDKAL1 | rs201339 | 21104291 | 0.0022578 | 0.98151 | 0.13667 | 0.044749 |
| PTPRD | rs10125894 | 8356757 | 0.0022693 | 1 | 0.14048 | 0.04602 |
| CDKAL1 | rs4710960 | 21047769 | 0.0023228 | 0.97926 | -0.13691 | 0.044955 |
| CDKAL1 | rs383684 | 21089414 | 0.002366 | 1 | 0.1355 | 0.044573 |
| HNRNPA3P1 | rs7920120 | 43627281 | 0.0024229 | 0.95049 | 0.20421 | 0.067332 |
| CDKAL1 | rs7747773 | 21046585 | 0.0024263 | 0.97434 | 0.13661 | 0.045049 |
| HNRNPA3P1 | rs1414491 | 43632364 | 0.0024916 | 0.93834 | -0.17761 | 0.058726 |
| PTPRD | rs12684548 | 9091611 | 0.0026652 | 0.43441 | 0.31923 | 0.10627 |
| HNRNPA3P1 | rs7080348 | 43626248 | 0.0026779 | 0.97203 | 0.2015 | 0.06711 |
| DMRTA1 | rs7858725 | 22463237 | 0.0027428 | 0.98037 | -0.13064 | 0.043617 |
| HNRNPA3P1 | rs7071492 | 43633738 | 0.0027482 | 0.92426 | -0.19392 | 0.064756 |
| DMRTA1 | rs16906193 | 22462796 | 0.0027748 | 0.98634 | 0.13 | 0.043456 |
| CDKAL1 | rs7768647 | 21042991 | 0.00278 | 0.96078 | 0.13551 | 0.045306 |
| DMRTA1 | rs7026813 | 22462338 | 0.0028179 | 0.99296 | -0.12928 | 0.043281 |
| TCF7L2 | rs12354626 | 114762419 | 0.0028271 | 0.36167 | 0.35102 | 0.11756 |
| DMRTA1 | rs17240142 | 22462208 | 0.0028288 | 0.99363 | -0.12917 | 0.043263 |
| DMRTA1 | rs17253318 | 22476246 | 0.0028403 | 0.93676 | -0.13424 | 0.044977 |
| DMRTA1 | rs7027027 | 22462289 | 0.0028406 | 1 | -0.12899 | 0.043221 |
| HNRNPA3P1 | rs1540967 | 43623936 | 0.0028826 | 1 | -0.19807 | 0.066468 |
| MTFHD1L | rs7743138 | 151542388 | 0.0029375 | 0.16277 | 0.51917 | 0.17456 |
| PTPRD | rs10977015 | 8364060 | 0.0030509 | 0.98449 | 0.12557 | 0.042386 |
| HNRNPA3P1 | rs11238721 | 43633162 | 0.003062 | 0.91655 | -0.19166 | 0.064719 |
| HNRNPA3P1 | rs7079939 | 43632112 | 0.0030774 | 0.90192 | -0.19257 | 0.065059 |
| HNRNPA3P1 | rs11238720 | 43633128 | 0.003087 | 0.91595 | -0.19151 | 0.064722 |
| ADAMTS9 | rs11130966 | 64456483 | 0.0031094 | 0.91347 | -0.1164 | 0.039369 |
| HNRNPA3P1 | rs12264669 | 43637054 | 0.0031958 | 0.97523 | 0.18774 | 0.063677 |
| HNRNPA3P1 | rs7086449 | 43637124 | 0.0032124 | 0.97577 | 0.18759 | 0.063661 |
| HNRNPA3P1 | rs4948579 | 43627030 | 0.0032848 | 0.9564 | -0.10776 | 0.036655 |
| DMRTA1 | rs17239912 | 22459860 | 0.0033402 | 0.99239 | -0.12658 | 0.043133 |
| TCF7L2 | rs11196192 | 114772277 | 0.003379 | 1 | -0.20033 | 0.068349 |
| HNRNPA3P1 | rs12266918 | 43638241 | 0.0034484 | 1 | 0.18475 | 0.063169 |
| DMRTA1 | rs7875770 | 22407161 | 0.0038265 | 0.98563 | 0.091298 | 0.031568 |
| DMRTA1 | rs16906157 | 22458301 | 0.0038764 | 0.99204 | -0.12421 | 0.043009 |
| MTFHD1L | rs2073191 | 151308257 | 0.0039981 | 0.71699 | 0.13409 | 0.046586 |
| DMRTA1 | rs616439 | 22453905 | 0.0041189 | 0.93186 | 0.12313 | 0.04292 |
| DMRTA1 | rs16906154 | 22457696 | 0.0041403 | 0.99211 | -0.12315 | 0.042949 |
| TCF7L2 | rs11196201 | 114793297 | 0.0042485 | 0.95475 | 0.20187 | 0.070605 |
| PTPRD | rs10976994 | 8351332 | 0.0045531 | 0.91027 | -0.13936 | 0.049121 |
| ST6GAL1 | rs1990676 | 188267332 | 0.0048281 | 1 | -0.12643 | 0.044861 |
| NCRNA00310 | rs2834885 | 35592140 | 0.004907 | 0.87992 | -0.099226 | 0.035273 |
| ADAMTS9 | rs9871177 | 64455825 | 0.0051726 | 0.89054 | -0.1116 | 0.039912 |
| TCF7L2 | rs12266632 | 114754949 | 0.0051771 | 0.9409 | 0.20224 | 0.072336 |
| DMRTA1 | rs7851527 | 22456171 | 0.0052833 | 1 | -0.11396 | 0.040856 |
| NCRNA00310 | rs2834892 | 35601241 | 0.0053575 | 0.98548 | 0.09362 | 0.033619 |
| PTPRD | rs10977557 | 9180404 | 0.005465 | 0.89149 | -0.098804 | 0.035563 |
| PPARG | rs4135263 | 12398266 | 0.005534 | 0.98629 | 0.11427 | 0.041191 |
| HNF1B | rs7407025 | 33154923 | 0.0056081 | 1 | 0.09291 | 0.033543 |
| PPARG | rs6809832 | 12389420 | 0.0057614 | 0.9827 | 0.11389 | 0.04125 |
| DMRTA1 | rs563802 | 22441031 | 0.0058264 | 0.90893 | 0.12055 | 0.043718 |
| NCRNA00310 | rs2834890 | 35599188 | 0.0060718 | 0.98859 | -0.091834 | 0.033469 |
| IRS1 | rs10192769 | 227408162 | 0.006138 | 1 | 0.09167 | 0.033452 |
| DMRTA1 | rs7024096 | 22389693 | 0.0061911 | 1 | 0.10444 | 0.038153 |
| IRS1 | rs2178702 | 227266168 | 0.0065308 | 0.96353 | 0.090827 | 0.033394 |
| MTFHD1L | rs4140529 | 151666429 | 0.0065525 | 0.92233 | -0.08817 | 0.03243 |
| NCRNA00310 | rs2142049 | 35601667 | 0.0065731 | 0.9786 | -0.091194 | 0.033555 |
| NCRNA00310 | rs2178817 | 35601700 | 0.0065803 | 0.97862 | -0.09118 | 0.033555 |
| SLC30A8 | rs6469670 | 118119171 | 0.0065989 | 0.87129 | 0.19432 | 0.071534 |
| DMRTA1 | rs17237814 | 22387161 | 0.0067295 | 0.97634 | -0.10443 | 0.038536 |
| HMGA2 | rs7973574 | 64558999 | 0.0068711 | 0.72768 | -0.20236 | 0.074863 |
| NCRNA00310 | rs2834893 | 35604155 | 0.0069329 | 0.98773 | -0.090518 | 0.033525 |
| MTFHD1L | rs6557548 | 150994462 | 0.0070132 | 1 | -0.12421 | 0.046068 |
| DMRTA1 | rs17835669 | 22444125 | 0.0070781 | 0.93076 | -0.12171 | 0.045193 |
| HMGA2 | rs11175992 | 64677663 | 0.0070962 | 0.64067 | -0.10704 | 0.039759 |
| IGLL1 | rs131428 | 22255431 | 0.0071135 | 0.65735 | 0.11478 | 0.042646 |
| NCRNA00310 | rs2834895 | 35604431 | 0.0071679 | 0.98846 | 0.090095 | 0.033506 |
| HNRNPA3P1 | rs10899916 | 43619326 | 0.0071712 | 0.96745 | -0.096443 | 0.035869 |
| HMGA2 | rs2272046 | 64510728 | 0.007173 | 0.79626 | -0.21134 | 0.078604 |
| DMRTA1 | rs11998892 | 22389009 | 0.0071964 | 0.98285 | -0.10308 | 0.038355 |
| HNRNPA3P1 | rs7916028 | 43625722 | 0.0072512 | 0.99582 | 0.093224 | 0.034719 |
| HNRNPA3P1 | rs10899917 | 43623622 | 0.0072867 | 0.99711 | -0.093125 | 0.034704 |
| DMRTA1 | rs7038201 | 22389391 | 0.0073006 | 0.98421 | -0.1028 | 0.038318 |
| HNRNPA3P1 | rs7922745 | 43623225 | 0.0073262 | 1 | 0.092799 | 0.034605 |
| HNRNPA3P1 | rs1537796 | 43624228 | 0.0073356 | 1 | 0.093027 | 0.034696 |
| HMGA2 | rs10400419 | 64676235 | 0.0074526 | 1 | -0.087497 | 0.032698 |
| GRB14 | rs10167297 | 165117962 | 0.0074758 | 0.99517 | 0.097437 | 0.036427 |
| DMRTA1 | rs1410978 | 22394681 | 0.0075587 | 0.93993 | -0.10588 | 0.039638 |
| KCNQ1 | rs231916 | 2704944 | 0.0076128 | 1 | 0.12621 | 0.04729 |
| C6orf105 | rs9296163 | 11900259 | 0.0077154 | 0.7453 | -0.099694 | 0.037419 |
| GRB14 | rs4667465 | 165120040 | 0.0077812 | 0.995 | -0.096843 | 0.036388 |
| CDKAL1 | rs374654 | 21117087 | 0.0077918 | 1 | 0.10189 | 0.038289 |
| DGKB | rs10241087 | 14240868 | 0.0079214 | 1 | -0.11939 | 0.04496 |
| CDKAL1 | rs4712569 | 21047359 | 0.007946 | 0.96979 | -0.10265 | 0.038672 |
| C6orf105 | rs4711430 | 11901775 | 0.0080008 | 0.75473 | 0.098601 | 0.037179 |
| ADAMTS9 | rs6803177 | 64447171 | 0.008004 | 0.73089 | 0.13475 | 0.050811 |
| GRB14 | rs11690448 | 165101603 | 0.0081933 | 0.99048 | 0.096532 | 0.03651 |
| LDLR | rs1799898 | 11088554 | 0.0085417 | 1 | -0.11105 | 0.042228 |
| GRB14 | rs3942459 | 165125480 | 0.0086604 | 0.99458 | 0.095248 | 0.036283 |
| PTPRD | rs17785914 | 9591885 | 0.0086943 | 0.98731 | 0.087223 | 0.033242 |
| GRB14 | rs1568504 | 165125650 | 0.0087509 | 0.99456 | 0.09509 | 0.036271 |
| ST6GAL1 | rs4686836 | 188212059 | 0.0088199 | 1 | 0.093266 | 0.035612 |
| ST6GAL1 | rs3872721 | 188208185 | 0.0088327 | 0.92061 | -0.095506 | 0.036474 |
| GRB14 | rs1106257 | 165103683 | 0.0088373 | 1 | 0.095101 | 0.036322 |
| MTFHD1L | rs275347 | 151012000 | 0.0089593 | 0.93597 | 0.12446 | 0.047622 |
| KCNQ1 | rs2237876 | 2721878 | 0.0090335 | 1 | -0.09173 | 0.035135 |
| CDKAL1 | rs9460610 | 21332272 | 0.0090625 | 1 | 0.11747 | 0.045015 |
| HNRNPA3P1 | rs2863760 | 43617585 | 0.009222 | 0.95179 | -0.093802 | 0.036026 |
| HNRNPA3P1 | rs4948779 | 43614521 | 0.0094916 | 0.98796 | 0.11196 | 0.043166 |
| JAZF1 | rs498475 | 28222765 | 0.0095214 | 1 | 0.080372 | 0.030999 |
| CD83 | rs1535359 | 14270208 | 0.0095523 | 0.69979 | 0.099002 | 0.038201 |
| GRB14 | rs12476305 | 165152920 | 0.0095845 | 0.87215 | -0.085702 | 0.033084 |
| CDKAL1 | rs415446 | 21140661 | 0.0096731 | 1 | -0.080286 | 0.031031 |
| HNF4A | rs4812833 | 42502410 | 0.0097625 | 1 | -0.079096 | 0.030608 |
