# Supplementary material for: Large Scale Association Analysis Identifies Three Susceptibility Loci for Coronary Artery Disease
Source: PLoS One. 2011 Dec 27;6(12):e29427. doi: 10.1371/journal.pone.0029427 (PMC3246490; doi:10.1371/journal.pone.0029427)
Supplement: Table S2 — Proportional Odds Logistic regression predicting CAD in graded categories from 10 SNPs. Proportional Odds Logistic regression predicting CAD in graded categories from 10 SNPs, both with additive and independent homozygous/heterozygous odds, without and with adjustment by family history of CAD, history of smoking, diagnoses of diabetes, hyperlipidemia, hypertension, and gender are represented. Odds ratio tests of disease vs. haplotype frequency are also indicated. (DOC) [file pone.0029427.s002.doc]

**Table S2.** **Proportional Odds Logistic regression predicting CAD in graded categories from 10 SNPs.**

| **Genotype** | **CAD cases % (n)** | **Controls % (n)** | **OR 95% CI** | **p-value** | **Adj. OR 95% CI** | **p-value** | **Pub OR** |
| --- | --- | --- | --- | --- | --- | --- | --- |
| ***rs1041981*** | 100 (1522) | 100 (426) | 0.869 | 0.198 | 0.867 | 0.201 |  |
|  |  |  | (0.70,1.07) |  | (0.69,1.08) |  |  |
| *AA* | (156) | (50) | 0.66 | 0.126 | 0.67 | 0.145 |  |
|  |  |  | (0.37,1.10) |  | (0.38,1.27) |  |  |
| *AG* | (622) | (166) | 0.956 | 0.764 | 0.942 | 0.694 |  |
|  |  |  | (0.37,1.10) |  | (0.70,1.23) |  |  |
| *GG* | (741) | (209) |  |  |  |  |  |
| *A* allele | 28.2 | 31.2 | 0.85 | 0.354 | - | - |  |
| *G* allele | 78.1 | 68.8 | (0.62,1.17) |  | - | - |  |
| ***rs11206510*** |  |  | 1.023 | 0.869 | 1.028 | 0.843 |  |
|  |  |  | (0.79,1.35) |  | (0.79,1.36) |  |  |
| *TT* | (1068) | (297) | 1.017 | 0.969 | 1.006 | 0.947 |  |
|  |  |  | (0.46,2.69) |  | (0.45,2.68) |  |  |
| *TC* | (413) | (114) | 0.988 | 0.978 | 0.97 | 0.941 |  |
|  |  |  | (0.43,2.66) |  | (0.42,2.63) |  |  |
|  | (39) | (14) |  |  |  |  |  |
| *T* allele | 84.0 | 83.7 | 1.006 | 1.000 | - | - | 1.15 |
| *C* allele | 16.0 | 16.3 | (0.68,1.51) |  | - | - | (1.10-1.21) |
| ***rs1746048*** |  |  | 1.079 | 0.520 | 1.084 | 0.501 |  |
|  |  |  | (0.86,1.36) |  | (0.86,1.37) |  |  |
| *CC* | (874) | (236) | 1.387 | 0.320 | 1.394 | 0.317 |  |
|  |  |  | (0.75,2.83) |  | (0.76,2.82) |  |  |
| *CT* | (546) | (157) | 1.390 | 0.328 | 1.389 | 0.333 |  |
|  |  |  | (0.75,2.83) |  | (0.74,2.85) |  |  |
|  | (96) | (31) |  |  |  |  |  |
| *C* allele | 76.6 | 75.2 | 1.080 | 0.680 | - | - | 1.17 |
| *T* allele | 23.4 | 24.8 | (0.77,1.53) |  | - | - | (1.11-1.24) |
| ***rs2048327*** |  |  | 0.896 | 0.389 | 0.916 | 0.499 |  |
|  |  |  | (0.69,1.15) |  | (0.71,1.18) |  |  |
| *CC* | (59) | (25) | 0.483 | 0.121 | 0.523 | 0.169 |  |
|  |  |  | (0.17,1.10) |  | (0.18,1.20) |  |  |
| *CT* | (492) | (129) | 1.017 | 0.914 | 1.03 | 0.849 |  |
|  |  |  | (0.75,1.38) |  | (0.76,1.39) |  |  |
|  | (971) | (271) |  |  |  |  |  |
| *C* allele | 18.7 | 20.5 | 0.908 | 0.658 | - | - |  |
| *T* allele | 81.3 | 79.5 | (0.52,1.30) |  | - | - |  |

**Table S2 (continued)**

| ***Genotype*** | **CAD cases % (n)** | **Controls % (n)** | **OR 95% CI** | **p-value** | **Adj. OR 95% CI** | **p-value** | | **Pub OR** |
| --- | --- | --- | --- | --- | --- | --- | --- | --- |
| ***rs3184504*** |  |  | 1.039 | 0.704 | 1.027 | 0.790 | |  |
|  |  |  | (0.85,1.26) |  | (0.84,1.25) |  | |  |
| *TT* | (296) | (83) | 1.026 | 0.903 | 0.993 | 0.975 | |  |
|  |  |  | (0.67,1.55) |  | (0.64,1.51) |  |  | |
| *TC* | (730) | (195) | 1.245 | 0.179 | 1.289 | 0.129 | |  |
|  |  |  | (0.91,1.72) |  | (0.93,1.80) |  | |  |
|  | (494) | (146) |  |  |  |  | |  |
| *T* allele | 44.1 | 43.2 | 1.040 | 0.829 | - | - | | 1.13 |
| *C* allele | 55.9 | 56.8 | (0.78,1.39) |  | - | - | | (1.08-1.18) |
| ***rs4977574*** |  |  | 1.354 | 0.0048 | 1.333 | 0.0086 | |  |
|  |  |  | (1.10,1.68) |  | (1.08,1.66) |  | |  |
| *GG* | (627) | (156) | 1.856 | 0.0117 | 1.837 | 0.0111 | |  |
|  |  |  | (1.17,3.07) |  | (1.14,3.09) |  | |  |
| *GA* | (685) | (195) | 1.390 | 0.188 | 1.421 | 0.166 | |  |
|  |  |  | (0.87,2.30) |  | (0.88,2.39) |  | |  |
|  | (208) | (72) |  |  |  |  | |  |
| *G* allele | 69.1 | 62.1 | 1.380 | 0.039 | - | - | | 1.29 |
| *A* allele | 30.9 | 37.9 | (1.01,1.90) |  | - | - | | (1.25-1.34) |
| ***rs646776*** |  |  | 0.829 | 0.151 | 0.833 | 0.169 | |  |
|  |  |  | (0.64,1.08) |  | (0.65,1.09) |  | |  |
| *TT* | (1090) | (292) | 1.133 | 0.794 | 1.196 | 0.710 | |  |
|  |  |  | (0.49,3.30) |  | (0.51,3.50) |  | |  |
| *TC* | (390) | (123) | 1.542 | 0.374 | 1.647 | 0.309 | |  |
|  |  |  | (0.65,4.56) |  | (0.69,4.89) |  | |  |
|  | (42) | (11) |  |  |  |  | |  |
| *T* allele | 81.8 | 84.4 | 0.84 | 0.379 | - | - | | 1.19 |
| *C* allele | 18.2 | 15.6 | (0.58,1.24) |  | - | - | | (1.13-1.26) |
| ***rs653178*** |  |  | 1.025 | 0.801 | 1.013 | 0.901 | |  |
|  |  |  | (0.84,1.25) |  | (0.83,1.24) |  | |  |
| *GG* | (297) | (85) | 1.002 | 0.992 | 0.966 | 0.875 | |  |
|  |  |  | (0.66,1.51) |  | (0.63,1.47) |  | |  |
| *GA* | (730) | (194) | 1.216 | 0.230 | 1.257 | 0.169 | |  |
|  |  |  | (0.89,1.68) |  | (0.91,1.75) |  | |  |
|  | (494) | (147) |  |  |  |  | |  |
| *G* allele | 43.9 | 43.3 | 1.036 | 0.829 | - | - | |  |
| *A* allele | 56.1 | 56.7 | (0.77,1.39) |  | - | - | |  |

**Table S2 (continued)**

| **Genotype** | **CAD cases % (n)** | **Controls % (n)** | **OR 95% CI** | **p-value** | **Adj. OR 95% CI** | **p-value** | **Pub OR** |
| --- | --- | --- | --- | --- | --- | --- | --- |
| ***rs6725887*** |  |  | 1.162 | 0.298 | 1.168 | 0.287 |  |
|  |  |  | (0.87,1.53) |  | (0.87,1.55) |  |  |
| *CC* | (31) | (4) | 1.349 | 0.542 | 1.386 | 0.509 |  |
|  |  |  | (0.45,3.24) |  | (0.46,3.36) |  |  |
| *CT* | (340) | (82) | 1.162 | 0.373 | 1.164 | 0.372 |  |
|  |  |  | (0.83,1.60) |  | (0.83,1.62) |  |  |
|  | (1151) | (339) |  |  |  |  |  |
| *C* allele | 14.2 | 12.4 | 1.190 | 0.391 | - | - | 1.17 |
| *T* allele | 85.8 | 87.6 | (0.77,1.79) |  | - | - | (1.11-1.23) |
| ***rs6922269*** |  |  | 0.718 | 0.0083 | 0.689 | 0.0035 |  |
|  |  |  | (0.56,0.91) |  | (0.53,0.88) |  |  |
| *AA* | (101) | (25) | 0.633 | 0.163 | 0.599 | 0.121 |  |
|  |  |  | (0.31,1.15) |  | (0.30,1.10) |  |  |
| *GA* | (563) | (153) | 0.661 | 0.0087 | 0.625 | 0.0037 |  |
|  |  |  | (0.48,0.90) |  | (0.45,0.854) |  |  |
|  | (850) | (246) |  |  |  |  |  |
| *A* allele | 19.8 | 25.6 | 0.726 | 0.082 | - | - | 1.09 |
| *G* allele | 80.2 | 74.4 | (0.50,1.04) |  | - | - | (1.05-1.14) |

Abbreviations: CAD, coronary artery disease; OR, odds ratio; CI, confidence interval; Adj, adjusted; Pub, published.
