# Supplementary material for: Large Scale Association Analysis Identifies Three Susceptibility Loci for Coronary Artery Disease
Source: PLoS One. 2011 Dec 27;6(12):e29427. doi: 10.1371/journal.pone.0029427 (PMC3246490; doi:10.1371/journal.pone.0029427)
Supplement: Table S3 — Logistic regression predicting MI from 10 SNPs. Analysis was performed both with additive and independent homozygous/heterozygous odds, without and with adjustment by family history of CAD, history of smoking, diagnoses of diabetes, hyperlipidemia, hypertension, and gender. Odds ratio tests of disease vs. haplotype frequency are also represented. (DOC) [file pone.0029427.s003.doc]

**Table S3. Logistic regression predicting MI from 10 SNPs.**

| **Genotype** | **MI cases % (n)** | **Controls % (n)** | **OR 95% CI** | **p-value** | **Adj. OR 95% CI** | **p-value** | **Pub OR** |
| --- | --- | --- | --- | --- | --- | --- | --- |
| ***rs1041981*** | 100 (222) | 100 (1727) | 0.869 | 0.198 | 0.867 | 0.201 |  |
|  |  |  | (0.70,1.07) |  | (0.69,1.08) |  |  |
| *AA* | (17) | (189) | 0.66 | 0.126 | 0.670 | 0.145 |  |
|  |  |  | (0.37,1.10) |  | (0.38,1.27) |  |  |
| *AG* | (91) | (698) | 0.956 | 0.764 | 0.942 | 0.694 |  |
|  |  |  | (0.37,1.10) |  | (0.70,1.23) |  |  |
|  | (114) | (836) |  |  |  |  |  |
| *A* allele | 28.2 | 31.2 | 0.85 | 0.354 | - | - |  |
| *G* allele | 78.1 | 68.8 | (0.62,1.17) |  | - | - |  |
| ***rs11206510*** |  |  | 1.023 | 0.869 | 1.028 | 0.843 |  |
|  |  |  | (0.79,1.35) |  | (0.79,1.36) |  |  |
| *TT* | (157) | (1209) | 1.017 | 0.969 | 1.006 | 0.947 |  |
|  |  |  | (0.46,2.69) |  | (0.45,2.68) |  |  |
| *TC* | (59) | (468) | 0.988 | 0.978 | 0.970 | 0.941 |  |
|  |  |  | (0.43,2.66) |  | (0.42,2.63) |  |  |
|  | (6) | (47) |  |  |  |  |  |
| *T* allele | 84 | 83.7 | 1.006 | 1.000 | - | - | 1.15 |
| *C* allele | 16 | 16.3 | (0.68,1.51) |  | - | - | (1.10-1.21) |
| ***rs1746048*** |  |  | 1.079 | 0.520 | 1.084 | 0.501 |  |
|  |  |  | (0.86,1.36) |  | (0.86,1.37) |  |  |
| *CC* | (129) | (981) | 1.387 | 0.320 | 1.394 | 0.317 |  |
|  |  |  | (0.75,2.83) |  | (0.76,2.82) |  |  |
| *CT* | (82) | (622) | 1.39 | 0.328 | 1.389 | 0.333 |  |
|  |  |  | (0.75,2.83) |  | (0.74,2.85) |  |  |
|  | (11) | (116) |  |  |  |  |  |
| *C* allele | 76.6 | 75.2 | 1.08 | 0.68 | - | - | 1.17 |
| *T* allele | 23.4 | 24.8 | (0.77,1.53) |  | - | - | (1.11-1.24) |
| ***rs2048327*** |  |  | 0.896 | 0.389 | 0.916 | 0.499 |  |
|  |  |  | (0.69,1.15) |  | (0.71,1.18) |  |  |
| *CC* | (5) | (79) | 0.483 | 0.121 | 0.523 | 0.169 |  |
|  |  |  | (0.17,1.10) |  | (0.18,1.20) |  |  |
| *CT* | (73) | (548) | 1.017 | 0.914 | 1.030 | 0.849 |  |
|  |  |  | (0.75,1.38) |  | (0.76,1.39) |  |  |
|  | (114) | (1099) |  |  |  |  |  |
| *C* allele | 18.7 | 20.5 | 0.908 | 0.658 | - | - |  |
| *T* allele | 81.3 | 79.5 | (0.52,1.30) |  | - | - |  |

**Table S3 (continued)**

| **Genotype** | **MI cases % (n)** | **Controls % (n)** | **OR 95% CI** | **p-value** | **Adj. OR 95% CI** | **p-value** | **Pub OR** |
| --- | --- | --- | --- | --- | --- | --- | --- |
| ***rs3184504*** |  |  | 1.039 | 0.704 | 1.027 | 0.790 |  |
|  |  |  | (0.85,1.26) |  | (0.84,1.25) |  |  |
| *TT* | (40) | (339) | 1.026 | 0.903 | 0.993 | 0.975 |  |
|  |  |  | (0.67,1.55) |  | (0.64,1.51) |  |  |
| *TC* | (116) | (810) | 1.245 | 0.179 | 1.289 | 0.129 |  |
|  |  |  | (0.91,1.72) |  | (0.93,1.80) |  |  |
|  | (66) | (574) |  |  |  |  |  |
| *T* allele | 44.1 | 43.2 | 1.040 | 0.829 | - | - | 1.13 |
| *C* allele | 55.9 | 56.8 | (0.78,1.39) |  | - | - | (1.08-1.18) |
| ***rs4977574*** |  |  | 1.354 | 0.0048 | 1.333 | 0.0086 |  |
|  |  |  | (1.10,1.68) |  | (1.08,1.66) |  |  |
| *GG* | (107) | (676) | 1.856 | 0.0117 | 1.837 | 0.0111 |  |
|  |  |  | (1.17,3.07) |  | (1.14,3.09) |  |  |
| *GA* | (93) | (787) | 1.390 | 0.188 | 1.421 | 0.166 |  |
|  |  |  | (0.87,2.30) |  | (0.88,2.39) |  |  |
|  | (22) | (258) |  |  |  |  |  |
| *G* allele | 69.1 | 62.1 | 1.38 | 0.039 | - | - | 1.29 |
| *A* allele | 30.9 | 37.9 | (1.01,1.90) |  | - | - | (1.25-1.34) |
| ***rs646776*** |  |  | 0.829 | 0.151 | 0.833 | 0.169 |  |
|  |  |  | (0.64,1.08) |  | (0.65,1.09) |  |  |
| *TT* | (146) | (1237) | 1.133 | 0.794 | 1.196 | 0.710 |  |
|  |  |  | (0.49,3.30) |  | (0.51,3.50) |  |  |
| *TC* | (71) | (442) | 1.542 | 0.374 | 1.647 | 0.309 |  |
|  |  |  | (0.65,4.56) |  | (0.69,4.89) |  |  |
|  | (5) | (48) |  |  |  |  |  |
| *T* allele | 81.8 | 84.4 | 0.840 | 0.379 | - | - | 1.19 |
| *C* allele | 18.2 | 15.6 | (0.58,1.24) |  | - | - | (1.13-1.26) |
| ***rs653178*** |  |  | 1.025 | 0.801 | 1.013 | 0.901 |  |
|  |  |  | (0.84,1.25) |  | (0.83,1.24) |  |  |
| *GG* | (40) | (342) | 1.002 | 0.992 | 0.966 | 0.875 |  |
|  |  |  | (0.66,1.51) |  | (0.63,1.47) |  |  |
| *GA* | (115) | (810) | 1.216 | 0.230 | 1.257 | 0.169 |  |
|  |  |  | (0.89,1.68) |  | (0.91,1.75) |  |  |
|  | (67) | (574) |  |  |  |  |  |
| *G* allele | 43.9 | 43.3 | 1.036 | 0.829 | - | - |  |
| *A* allele | 56.1 | 56.7 | (0.77,1.39) |  | - | - |  |

**Table S3 (continued)**

| **Genotype** | **MI cases % (n)** | **Controls % (n)** | **OR 95% CI** | **p-value** | **Adj. OR 95% CI** | **p-value** | **Pub OR** |
| --- | --- | --- | --- | --- | --- | --- | --- |
| ***rs6725887*** |  |  | 1.162 | 0.298 | 1.168 | 0.287 |  |
|  |  |  | (0.87,1.53) |  | (0.87,1.55) |  |  |
| *CC* | (5) | (30) | 1.349 | 0.542 | 1.386 | 0.509 |  |
|  |  |  | (0.45,3.24) |  | (0.46,3.36) |  |  |
| *CT* | (53) | (369) | 1.162 | 0.373 | 1.164 | 0.372 |  |
|  |  |  | (0.83,1.60) |  | (0.83,1.62) |  |  |
|  | (164) | (1327) |  |  |  |  |  |
| *C* allele | 14.2 | 12.4 | 1.190 | 0.391 | - | - | 1.17 |
| *T* allele | 85.8 | 87.6 | (0.77,1.79) |  | - | - | (1.11-1.23) |
| ***rs6922269*** |  |  | 0.718 | 0.0083 | 0.689 | 0.0035 |  |
|  |  |  | (0.56,0.91) |  | (0.53,0.88) |  |  |
| *AA* | (11) | (115) | 0.633 | 0.163 | 0.599 | 0.121 |  |
|  |  |  | (0.31,1.15) |  | (0.30,1.10) |  |  |
| *GA* | (65) | (651) | 0.661 | 0.0087 | 0.625 | 0.0037 |  |
|  |  |  | (0.48,0.90) |  | (0.45,0.854) |  |  |
|  | (144) | (953) |  |  |  |  |  |
| *A* allele | 19.8 | 25.6 | 0.726 | 0.082 | - | - | 1.09 |
| *G* allele | 80.2 | 74.4 | (0.50,1.04) |  | - | - | (1.05-1.14) |

Abbreviations: CAD, coronary artery disease; OR, odds ratio; CI, confidence interval; Adj., adjusted; Pub, published.
